# 

**Published:** 1874-04

**Authors:** 


### CONTENTS.

PAGE.

**CONTRIBUTIONS.**

Workday Playdays. BEN H. PRATT, A. M. ........ 219
Personal Beauty. P. H. HAYES, M. D. ........ 221
Patent Medicines. M. S. BIDWELL ........ 224

**CLIPPINGS.**

How to Predict the Weather ........ 225
Tea Drunkards ........ 225
Scotch Humor ........ 225

**MEDICAL ITEMS AND NEWS.**

Burns—Epithelioma of the Eye--Adhesive Plaster Chronic Eczema--Acute Articular Rheumatism—Irregular action of heart—Hydrocele—Ether—Chronic Synovitis — Cough Mixture — Chronic Hoarseness--Erysipelas--Night Sweats in Phthisis—Pneumonia — Morbus Brightii — Nocturnal Pains--Urine Tests—Angina Pectoris--Ringworm --Aromatic Candles--Subcutaneous injections in 1664--Codliver oil compound--Strumous Children—Peri and Endo Carditis, etc.—Caoutchouc—Neuralgia in Infants—Ergot in Epistaxes—Erysipelas--Surgical Dressing—Pneumatic Aspiration in Hydrarthrosis--Nit. Amyl--Inhalation of Vapors of Oxygenated Essences—Mint in suppression of Milk--Atropia in Phthisical Sweating --Delirium Tremens--Tape Worm ........ 226 to 233

PAGE.

**OUR CORRESPONDENCE.**

Seven Letters and Replies ........ 233

**HOUSEHOLD HINTS AND RECIPES.**

To protect drawings—Caustics vs. Weeds—Waterproofing Boots--Infantile Thumb Sucking--Hay Asthma -- Diabetic Bread -- Perpetual Paste--Cure for Baldness--Eating When Sick--Grease Eraser--Cement—Fuel--Sunshine and Sleep--Corns--Foul Ulcers--Flies on Horses ........ 235 to 236

**EDITORIAL.**

The Prevailing Excitement ........ 237
The Eye-Lids ........ 239
The Ophthalmoscope ........ 240
A Slight Controversy on Antipathy of the Sexes ........ 241

**CHIT-CHAT.**

A Bull—Too Bad--Fire—A Struggle--Fashion--Quack Medicine--Rough Accusations--Law and Medicine--Insane--Clever of Them--Roller Towels—Fastidious--Good Law--Read It--Twins--Ten Years Editorship--The Women--Massachusetts Petition--Railway Accidents--Patent Medicine Proprietors—Better Late than Never--Ye Olden Time--Signs of the Times--Delay--Let's go Fishing ........ 243 to 245

